# Social and material aspects of life and their impact on the physical health of people diagnosed with mental illness

**DOI:** 10.1111/hex.12539

**Published:** 2017-03-15

**Authors:** Stephanie B. Ewart, Brenda Happell, Julia Bocking, Chris Platania‐Phung, Robert Stanton, Brett Scholz

**Affiliations:** ^1^ SYNERGY: Nursing and Midwifery Research Centre University of Canberra and ACT Health Canberra Hospital Woden ACT Australia; ^2^ School of Medical and Applied Sciences Central Queensland University Rockhampton QLD Australia

**Keywords:** mental health, mental illness, physical health, social determinants of health

## Abstract

**Background:**

People diagnosed with mental illness have shorter lives and poorer physical health, compared to the general population. These health inequities are usually viewed at an individual and clinical level, yet there is little research on the views of mental health consumers on clinical factors in broader contexts.

**Objective:**

To elicit the views of consumers of mental health services regarding their physical health and experiences of accessing physical health‐care services.

**Design:**

Qualitative exploratory design involving focus groups.

**Setting and participants:**

The research was conducted in the Australian Capital Territory. Participants were consumers of mental health services.

**Main outcome measures:**

The Commission on Social Determinants of Health Framework was drawn on to lead deductive analysis of focus group interview transcripts.

**Results:**

Issues impacting consumers included poverty, the neglect of public services and being treated as second‐class citizens because of diagnosis of mental illness and/or experiencing a psychosocial disability. These factors were connected with significant barriers in accessing physical health care, including the quality and relevance of health provider communication, especially when the broader contexts of mental health consumer's lives are not well understood.

**Discussion and conclusions:**

These findings suggest the Commission on Social Determinants of Health Framework could be utilized in research and policy, and may provide an effective platform for exploring better health communication with mental health consumers regarding this neglected health inequity.

## 1 INTRODUCTION

It has been found internationally that people diagnosed with mental illness live around 20 years shorter lives than the general population.^1^, ^2^, ^3^ The earlier mortality of people with mental illness has been linked to higher rates of physical illnesses, such as coronary heart disease.^4^, ^5^, ^6^ In addition, iatrogenic impacts on physical health such as the negative interaction of antipsychotic medication on metabolic processes have been consistently found.^7^ The inequalities in physical health between people with and without mental illness has been the focus of health‐care sector reforms such as integrated care models^8^ although large‐scale and decisive approaches are yet to be demonstrated.

In focusing on health services, behaviours and clinical practice, there is the risk of oversight of social–economic conditions such as employment, housing, transport and safety.^9^, ^10^ These factors may heavily impact the overall health of mental health consumers, and are therefore relevant to illness prevention, management and recovery. Socio‐economic forces are also associated with increased risk of physical illness and mental illness,^11^, ^12^ but usually only very briefly referred to as contributors to inequalities in physical health for people with mental illness, for example.^13^ Consistent with international literature on the public health issue of co‐occurrence of physical and mental illness,^14^ much research focuses primarily on population patterns in risks, service patterns and clinical data. In the largest available study of people with psychosis in Australia, for instance, socio‐economic factors were found to have impacted on a consumer's physical health status^15^ (p. 709). Study participants were asked to nominate their perceived “challenges in the next year.” In decreasing order of importance, participants were affected by “financial matters,” “loneliness/social isolation,” “lack of employment” and “poor physical health/physical health issues,” “uncontrolled symptoms of mental illness” and “lack of stable/suitable housing.” It is interesting to note that most of these concerns are only minimally looked at in the research literature on physical health inequalities.

The Commission on Social Determinants of Health, active between 2005 and 2008, did do some work^16^, ^17^ towards improving understandings of the types of economic, social and cultural problems raised in the study by Carr et al.^15^ A body of research literature was developed corresponding to the work of the commission, for example see.^18^ Both the commission and the research work report on associations of health with inequalities in housing, income, occupation level, education, safety, transport infrastructure, social services, water and agriculture.^9^, ^10^, ^19^

Solar and Irwin^20^ provided an original consolidated schema for The Commission on Social Determinants of Health (herein referred to as the CSDH Framework). In the CSDH Framework, health equity is affected by two types of determinants.^20^ The most proximal set of determinants of health are described as the “intermediary determinants of health” and comprise “material circumstances,” “behaviours and biological factors” and “psychosocial factors”^20^ (p. 6). These intermediary determinants also include the “health system,” which interacts with the above, mediating (in either positive or negative ways) their impact on health equity. Importantly, the broadest set of determinants in the framework is called the “structural determinants of health *inequities*.” In the CSDH Framework, the “socioeconomic and political context” (governance, macroeconomic, social and public policies and cultural and societal values) has bidirectional relationships with “socioeconomic position” (including social class, gender, income, occupation and education)^20^ (p. 6). The WHO^16^ (p. 7) explains that these broader “structural determinants” are the most salient determinants as they produce stratification within society (including via “discrimination on the basis of factors such as gender, ethnicity or disability”). The resulting health inequities then shape the aforementioned intermediary determinants of health.

The limited research literature articulating mental health consumers’ views regarding mental health service responses to physical health issues suggests that immediate physical health problems could not be divorced from appreciation of the social and material context of daily lives.^21^, ^22^ Given that the areas presented in the CSDH Framework resonate with such views, it is important to look more deeply into mental health consumers’ views on these broader factors, and how they relate to access to the health‐care system and overall states of health.

### 1.1 Aim

The current research is a qualitative exploratory study of mental health service consumers’ perspectives of physical health, including their experiences of interacting with health‐care providers, and their views with respect to impediments to, and enablers of, physical well‐being. In particular, in reporting on consumer perspectives, this paper explores the pertinence of the CSDH Framework to mapping out the views of people with mental illness.

## 2 METHOD

The research project adopted a qualitative approach to facilitate participants’ open sharing of their experiences and opinions as expert informants on the topic of investigation.^23^ This approach has been used to provide an in‐depth examination of the views of mental health consumers on topics where little work has been previously published.^24^

### 2.1 Participants

The researchers collaborated with the ACT Mental Health Consumers Network (ACTMHCN) to conduct this research. The ACTMHCN is the peak mental health consumer‐run public organization in the region, with an emphasis on the rights of people with mental health difficulties, social justice, policy reform and advocacy. The study was advertised through the ACTMHCN membership bulletin. Thirty‐one members participated in one of four focus groups. All participants were over the age of 18, and there were male and female participants in each of the focus groups.

### 2.2 Data collection

All focus groups were conducted at the ACTMHCN office, which provided an accessible, central location and familiar environment for prospective participants. Two interviewers conducted the four focus groups together: one an experienced academic in mental health nursing; the other, a consumer researcher with advocacy expertise and substantial interest in physical health. Focus groups were recorded via electronic audio devices with the consent of participants. The duration of focus group ranged from 90 to 150 minutes. Some broad and general questions were asked, designed to elucidate views and stories about physical health and service responses to their normative and expressed needs. For instance, the questions centred on participants’ views on their experiences with health‐care services in accessing treatment for physical health issues; participants were strongly encouraged to introduce any issues deemed relevant. The partnership with a consumer researcher was intended to be conducive to open discussions.

Focus groups were conducted until no new information was forthcoming (known as data saturation). Prospective participants were provided four focus group session times. After the third focus group, it was deemed that saturation was reached (no new topics or issues were being raised by participants). It was decided that the fourth focus group that had been earlier scheduled still proceed, with the purpose of providing more depth to what had been revealed in the first three group interviews and retain the opportunity for members of the consumer organization to voice their views.

### 2.3 Ethics

The research project proceeded after formal approval was granted by the university Human Research Ethics Committee. The voluntary basis of participation was stressed in the invitation to the study, and at the commencement of each focus group. At this point, participants were also provided assurance of the confidentiality of responses. This was a small‐scale and local study and participants were all members of a small organization, which presented heightened ethical issues with respect to data collection and the potential for identification^25^ It was crucial to assure prospective participants that data and reporting would preserve confidentiality. Reporting of subject background such as gender and age could compromise confidentiality and was therefore not recorded. The researchers also reiterated that participation would not affect their current or future mental health care. Each participant was reimbursed $75 for their time and expenses, as per ACTMHCN guidelines which align with ACT Government and Commonwealth of Australia policy.^26^, ^27^

### 2.4 Data analysis

Despite the predominance of inductive methodology in qualitative research, deductive analytical techniques informed by the social sciences can sensitize health researchers to conceptual insights that they might not otherwise have identified.^28^ MacFarlane and O'Reilly‐de Brun^28^ (p. 616) have argued that “orienting concepts need to be critically interrogated” and this slightly revised CSDH analytical framework opened a space for capturing consumer commentary on their experiences of mental health‐related disability and disability discrimination. Accordingly, the data analysis was conducted with NVIVO10, and all coding was pre‐set on the latest conceptual framework for action on the social determinants of health^20^ designed for the CSDH WHO.^17^ “Disability” (and related discrimination) was added, given this concept was specifically mentioned by the WHO,^16^ and deemed as conceptually meaningful to the topic of the physical health of mental health consumers. Initial data analysis was undertaken by a member of the research team who had co‐facilitated focus groups. Data analysis was led by the consumer researcher. A clear and rigorous process was followed. Once the initial coding had been completed, the data were independently reviewed by two other members of the research team, including one member who had not been part of the focus groups. The analysis was further refined until consensus was reached.

After in‐depth discussion of the initial coding by one of the focus group interviewers, respective researcher interpretations, other members of the research team further refined the analysis, including the other co‐facilitator of the groups.

## 3 RESULTS

Although this research study was oriented to the health service context, a striking (unprompted) emphasis in all focus groups was the salience of social and economic and discriminatory conditions in mental health consumers’ lives that had considerable impact on both their physical and overall health. In keeping with the deductive approach, participant responses will be reported as part of the CSDH Framework. The focus group (FG) number is indicated in each instance of a quote.

### 3.1 Structural determinants: Socioeconomic political context

We begin with the broadest layer of the CSDH Framework, which includes governance, macroeconomic policies and social and public policies. Participants oriented to the importance of government infrastructure and supports, including promotion of national public and civic organizations that are directly committed to mental health consumers. It was argued that there was a decision to abandon funding and many years of groundwork and momentum had been lost:

…we can't even get [Federal] funding from the politicians to fund our national consumer peak body that we've had all this planning for over the last six or eight years and with the Department of Health … we had the model and the constitution all ready to go, but they've refused to fund it now in the politics of, oh, we don't want to. (FG4)

Participants brought up personal difficulties and the need to connect these with affordability of utilities such as power and water, and the need for government allowances and social housing:

It's also the fact that we're on a disability pension and even if you're in housing, half—literally half of it goes to electricity and bloody housing and thank God gas is cheap because I—but, so, yeah, [sigh]. (FG4)

Participants spoke about the interface between public policy and the individual in relation to education, health and social protection. For instance, one participant recounted a very stressful experience of uncertainty regarding continuation of the disability support pension:

…every three months I have to go in for a Centrelink [Government Department administering welfare payments] appointment…the day that I get the notice…to literally the minute of my Centrelink appointment I am just becoming more and more anxious and more and more angry and…thinking they're going to take my pension… I'm not going to have enough food and then when I'm on the streets, I lose Centrelink [payments] because I won't have an address… (FG4)

Responses suggested that the basis for genuine communication between consumers and government services was not apparent, as more fundamental issues were not addressed, when people are evaluated in the light of social pressures borne of political rhetoric:

I trust[services] to step on me and crush me and try and break me into a bloody zombie, yes, sir, I will get a job and eat correctly… so there's all that sub‐culture side, that social pressures that feed into this too around the pressures to be seen as—not as—a lifter, not a leaner and all this rhetoric that seems to be really heightening these days. (FG4)

Labelling and compartmentalizing mental health consumers was noted as being a barrier to mental health consumers been seen as full citizens:

I think getting rid of a lot of that stuff would really help just open up the conversation, and just stop this physical health, mental health consumers…. this compartmentalising. Like, we're all part of the—we're communities…we're citizens, but nothing good comes from labelling like mental health issues or….physical health issues. That labelling, I think, is just really counterproductive. (FG1)

Another participant provided a specific example of negative social attitudes and judgements about people with mental illness:

…and I think in general, society think[s], oh, well, that person is just taking up a hospital bed because they're not really sick, they're just being manipulative and it is really a mental illness where they can't control it, so, yeah, there is judgement around that too. (FG4)

Each focus group had discussed the common experience of weight gain if using psychiatric drugs in efforts to ameliorate the impacts of mental health problems. Part of a sense of lower perceived worth also arose from societal weight‐based discrimination where people were not aware of the adverse effects of antipsychotic medication:

I don't think it goes through their head that maybe it's a side‐effect of medication and, so I think there is a stigma with being overweight by what other people in our society perceive as our motivations to be and I think they feel we're worthless as people if we're overweight. (FG4)

### 3.2 Structural determinants: Socioeconomic position and social stratification

People's lower economic position came up in many ways, such as the multiple difficulties of meeting living costs. Affordability of health care was an issue, not necessarily addressed by bulk‐billing [a process where the medical centre claims the government rebate and there are no out of pocket expenses for the consumer]:

Even if they do bulk bill, if they say the only way to treat this is with a medication that's non PBS [The PBS or Pharmaceutical Benefits Scheme is the major Australian government subsidy, for increased community access to prescription medicines] or what you need to do is go to this service, which is a fee for service thing, where if you don't have private health insurance you're paying full cost. So if going to the GP for any reason is actually quite terrifying for me, because I'm going to walk away being told that the only way you can fix this is to do this thing that you can't afford. (FG2)

There was some mention of violence and experiences of trauma were raised by several participants:

There's also the precursors to all of this stuff which is the family abuse: physical, sexual, mental abuse by the family and other members, and neglect … Which is happening a lot. (FG3)

In terms of ethnicity, a participant questioned whether the physical health‐care needs of particular culturally and linguistically diverse communities were receiving adequate attention:

And then there's the multicultural group—who's looking after physical health of the people from the multicultural area who've got mental illness? (FG4)

Furthermore, being employed was not seen as necessarily enriching and often as alienating, where people were not valued:

And they want me to go and throw myself into a group of complete strangers that want nothing to do with me really except for me to do my job and want me to focus for eight hours a day, you know what I mean? It's just not feasible and it's not practical… (FG4)

Participants talked in detail about diverse experiences of disability. These included profound energy loss and physical immobilization, intense fears (which could prevent people leaving home), suicidality, cognitive overload (such as distraction from hearing distressing voices), intense mood states (depression, mania), overwhelming physical agitation. In addition, many provided examples of direct psychosocial disability related to mental health states, such as social withdrawal, loss of self‐esteem and loss of ability to carry out basic daily tasks:

…mental illness…it does affect your motivation and your ability to even get yourself out of the house to even make, let alone—or the cognitive disabilities that can sometimes prevent you from making a phone call or an appointment. (FG4)

### 3.3 Intermediary determinants

The “intermediary determinants” raised were “lifestyle” behaviours in the context of issues of social responsibility and material factors. Participants saw particular behaviours as important contributors to health, but indicated that the entire responsibility had been transferred by the health system to the consumer to address all on their own:

Of course, the list of, are you walking; are you eating fresh fruit and vegies; are you doing this, are you doing that? And then it put it back onto myself. Okay. Well, I need to go off and do this. But when you're at home and then you're trying to get energy and the price of these goods these days are more expensive than getting some processed food, it's back onto the shoulders of the mental health consumer, being passed by the practitioner. (FG2)

A lack of economic resources directly impacted on the feasibility to do activities important to maintaining health, such as recreational walking. This issue is exemplified by the following:

Poverty is just—poverty actually stops me from going for walks as well, because I don't like to go for walks if I—because I still can't afford a mobile phone … in case I fall over or something, and I need to call someone. (FG1).

Infrastructure was also discussed in terms of access to health care, where transport loomed as a constant barrier for participants. For instance, one participant stated:

…..transport is a big issue. Especially when you have a spread out town like Canberra, you've got to rely on coordinating timetables for buses and everything else, let alone trying to run a car or pay for it and parking, and it's just getting worse…and so it becomes a vicious circle. (FG4)

Alongside transport, poverty also affected using public services that may be deemed “accessible and free” but where issues of poverty and rules would lead to more economic barriers:

But I don't see why I should… have to go to the library to access the Internet…I could go there, but I actually have a library fine. I have this poverty. (FG1)

Apart from logistics of accessing health and other public services, the whole sense of place was seen as negative due to issues of safety and a sense of despondency:

And it just impacts on my mood, and I just feel really crappy and living in a dodgy public housing environment…of drug addicts and alcoholics and abusive individuals that get transferred into my area because they can't live anywhere else…. But it's this whole…environment as well as my own physical and mental health that's—just doesn't seem to be a solution to it. (FG1)

Specific behaviours were discussed in relation to physical health such as physical activity, eating and smoking. Smoking was seen as highly prevalent yet there was no screening for lung‐related illnesses:

…and there'll be so many things going on out there, how many—65% or something of people with mental health smoke, do we regularly give them chest x‐rays looking for emphysema? No. (FG4)

Psychosocial factors were a major area. A participant suggested that physical health be seen as interpersonal and connected to other humans:

I mean, I'm low‐income. I find that being on a really low‐income and being by myself all the time, I also don't have any physical contact with other human beings in any way, and that's another unhealthy—that's another thing—in good health, for people to touch—human touch with other people. (FG1)

Another fundamental social dimension was place and others as source of purpose and meaning—this might involve not being at home all the time and visiting the Central Business District (CBD) of the Australian Capital Territory (ACT):

Yeah, it should be an easy walk into (ACT CBD) but I hardly ever walk in here [other participant: …No, but it's not just (ACT CBD), it's, as you said, a purpose, somewhere to go. So you need psychosocial—and socialisation around other people….

### 3.4 The health‐care system

Many participants spoke of health‐care access being severely constrained by poverty:

And in our (member) survey that we recently (conducted)… people raised in terms of things that were issues for them personally, the second one was cost of services… (FG3)

There were also discussions in each group about judgemental or dismissive health provider communication, diagnostic overshadowing and even outright experiences of discriminatory comments whilst seeking physical health care as a mental health consumer:

…and so that got [ a general practitioner's] back up a bit and there was tension. And I pointed out to the doctor, “Look, he's got a mental illness.” He said, “I don't want people with mental illness here.” (FG1)

A lack of support with unwanted impacts of psychiatric medication was another issue for participants. Lack of communication occurred when the provider lacking knowledge on what the medications could do to physical health:

That's what really bugs me. There's a list of side‐effects and you're, like, okay, could this possibly a side‐effect of the medication? And they say no, I'm, like, “Do you know all the side‐effects of this medication or… which a lot of time they don't (FG4)

## 4 DISCUSSION

Participants emphasized how the social, economic and material aspects of their lives were impacting on their physical, mental and overall health. Indeed, the emphasis on social and material factors was striking, given that literature and policy on inequalities in physical health of people with mental illness tends to consider “social–economic” factors in passing, and instead, takes a heavily individual and clinical approach to the issues at hand, for example.^14^ It was also notable that in this brief deductive secondary analysis, how much of the CSDH Framework^20^ was relevant to the physical health issues pertinent to consumers.

What is particular to the CSDH Framework is the emphasis on the health‐care system itself as a potential determinant of health.^20^ Participants described the health system as contributing to worse health outcomes—where lack of communication about the side‐effects of psychiatric drugs, negative staff attitudes and an overall lack of support were all concerns of consumers. Such views are not dissimilar to those aired by mental health consumers in other parts of Australia and internationally.^22^, ^29^ Consumers frequently talked about weight gain and medication, but did not describe routine screening or demonstrate awareness of metabolic health. No consumers reported receiving regular metabolic monitoring despite a number of participants having experienced taking medications that they reported as having caused them to gain weight. The consumers’ views were indeed consistent with health provider views on professional neglect of physical health issues.^30^ A final point on the interface of the health‐care system with broader society^20^ is how participants felt they were treated as second‐class citizens (or, indeed, as non‐citizens). It is concerning that, to participants, neglect by the health‐care system was a manifestation of stigma and unfair judgement by the wider society. These findings affirm the need to continue to challenge the stigma of mental illness, which continues to be a significant problem in Australia and internationally.^31^, ^32^, ^33^

The principles of patient and public involvement and engagement (PPIE) are recognized as internationally as integral to rigorous health‐care research.^34^ The current study reflects these principles^35^ Service user‐led research design and implementation is a significant component of PPIE.^36^ A consumer researcher was centrally involved in all phases of the current study—from co‐leading the framing of the research and development of the design and methods (eg focus group questions), to co‐facilitating focus groups, and leading data analysis and manuscript preparation.

The consumer researcher contributed her expertise and experience in all aspects of the collaborative research process, in accordance with the Principle of Practice of “Researchers, research organisations and the public respect one another's roles and perspectives”^35^ (p. 5). It is essential with PPIE that “Researchers, research organisations and the public have access to practical and organisational support to involve and be involved”^35^ (p. 6). In this study, the consumer researcher was employed by the research organization and actively supported in terms of research supervision and access to resources to fulfil major research activities and therefore have the opportunity to become an active member of the research team.

The consumer researcher was strongly committed to all aspects of the research study. The presence and influence of the consumer researcher in the focus groups was important to enabling a more open discussion than may have been possible. This provides evidence for principles in practice 4c that “Public members commit to their involvement in research and are willing to contribute to the research” (p. 9). In addition, inclusion of consumer researchers ensures that all steps in investigation are more geared to the consumer‐orientated service approach that is espoused in health policy.^32^, ^37^

A limitation of the present study was that participants were not asked for demographic information (eg gender, income, social security status), with the researchers relying on comments in the focus groups to understand these factors. Nonetheless, the research objective was to understand consumers’ subjectivities of their experiences. The major economic difficulties that participants oriented to are likely to be more salient to consumers’ lives than measures of income and welfare benefits received. Consumer views indicate that poverty has a profound effect on health‐care access, foregrounding the importance of wholly government‐funded health coverage and its importance for mental health consumers’ physical health outcomes. Australia is moving away from truly universal health care with increasing co‐payments and private sector involvement. The findings of the current study raise questions on whether such changes would disproportionately affect mental health consumers trying to maintain their physical health and longevity.

Efforts to reduce inequalities in physical health need to go beyond the current foci of the literature and Australian health policy (largely service provision and lifestyle education and interventions) to address social economic and material forces of everyday life, such as social isolation, and the wider society. The CSDH Framework should be explored as a platform for redirecting communication between stakeholders, to more robustly address the premature death and worse physical health of people with mental illness in Australia and internationally. These findings may encourage more research into whether mental health‐related disability—and specifically, the ways in which a range of government policies and broader social and cultural (mis)perceptions produce socioeconomic stratification and discrimination—may be a critical structural determinant of the physical health inequities for mental health consumers. Mental health consumer leaders and health‐care providers will play an important role in transferring the CSDH into current debates on inequalities in health.
